# Preliminary Physical and Chemical Characterization of By-Products from Cuban Coffee Production

**DOI:** 10.3390/foods13213348

**Published:** 2024-10-22

**Authors:** Dayana Mesa, Juan P. Figueroa, Eduardo A. Leyes, Carlos R. Castillo, Amanda Collazo, Harold A. Núñez, Dayamí Viltres, Yaneris Mirabal, Yamilet Coll

**Affiliations:** 1 Center for Natural Product Research, Faculty of Chemistry, University of Havana, Zapata and G, Havana 10400, Cuba; dayana.mesa@fq.uh.cu (D.M.); juan.figueroa-macias@mtl.maxplackschools.de (J.P.F.); eduardoleyes73@gmail.com (E.A.L.); crcastillossp@gmail.com (C.R.C.); acollazoaldana@gmail.com (A.C.); agusto71@gmail.com (H.A.N.); 2 Instituto de Investigaciones Agroforestales Cruce de los Baños, Santiago de Cuba 92700, Cuba; dayamibarbanviltres@gmail.com; 3 Faculty of Engineering, Institute of Applied Chemistry, Universidad Autónoma de Chile, Talca 3460000, Chile

**Keywords:** residue, coffee husk, coffee parchment, spent coffee grounds, characterization, utilization

## Abstract

Coffee is one of the most consumed beverages worldwide. Its production generates a large amount of waste, and its use is of vital importance to prevent it from becoming a source of environmental pollution. Cuba is a country with a well-known coffee-growing tradition. Although coffee production has decreased, it is vitally important to use the waste generated in these productions to reduce environmental pollution. To know the possible use or application of coffee waste, it is necessary to know its composition. In this article, three Cuban Arabica coffee wastes (husk, parchment and spent coffee grounds) were characterized using chemical, physical and physicochemical methods. In the characterization of these wastes, SEM and EDX were used to determine their microscopic form and chemical composition. The Chesson–Datta method, ATR and TGA were used to determine whether these materials were lignocellulosic. Ash, pH and density of the waste were determined as characterization methods. The extractive content was determined and a phytochemical screening was performed to determine the groups of the secondary metabolites present.

## 1. Introduction

**Figure 1:**
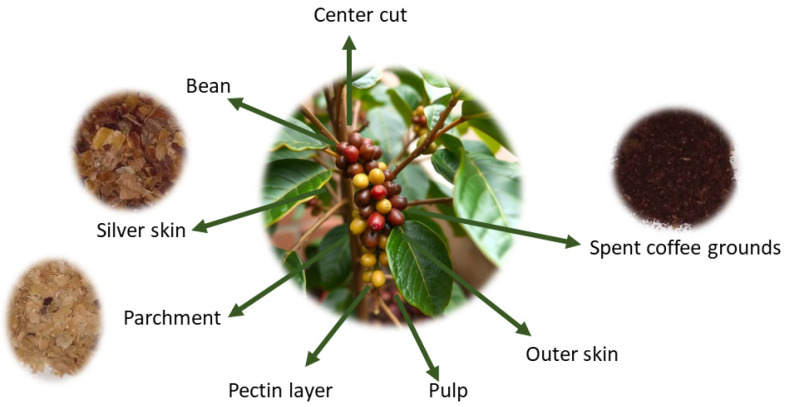
Coffee production-related by-products.

Coffee is one of the most consumed beverages worldwide and is, after oil, the second most traded product [1]. The global coffee production in the 2022/2023 agricultural season was approximately 168.2 million bags [2]. However, the coffee industry generates large amounts of toxic solid waste, at least 6–8 million tons worldwide per year [3], which can potentially cause serious environmental problems [4]. These residuals consist of husks, pulp, mucilage, silver skins, flowers, leaves, twigs, wood and coffee grounds, which are derived from the different stages of coffee processing, including harvesting, processing, roasting, and brewing the beverage [5,6] (Figure 1).

Among these waste and by-products, spent coffee grounds are the most abundantly generated during processing (650 g per kg of fresh coffee cherry), while parchment and coffee husks represent only between 35–61 g and 120–180 g per kg of fresh coffee cherry, respectively [7]. Given the fact that most of these residues are piled up or burned out, their accumulation could be considered an environmental hazard due to CO_2_ emission during burning or the pollution of the soil by the lixiviation of toxic secondary metabolites; hence, additional procedures are needed to tackle this problem in a sustainable manner [8].

Coffee residues contain a variety of components such as cellulose, hemicellulose, lignin, ashes, minerals, fats, alkaloids, proteins, and a combination of dietary fibers, among others. These residues can be used in versatile applications such as the extraction of bio components and colorants; as part of building materials; and the production of liquors, baked goods and food preservatives, among others. Beyond these applications, its potential as a precursor for polymers/composites, catalysts, and as a nutrient-rich feed or food ingredient has been recognized due to its abundant nutrient content [6,7,9].

In Cuba, coffee has been an important part of everyday life since the early XIX century, when commercial coffee production boomed in the island after the Haitian revolution [10]. From the island, around 1.5 tons per year of Arabica coffee is exported worldwide, standing out for its recognized quality [10]. Nevertheless, until now, the waste and by-products generated by the Cuban coffee industry have been severely underutilized. Therefore, the aim of the present work was to conduct a physical and chemical preliminary characterization of husks, parchment and spent coffee grounds, i.e., the by-products from Cuban production. This knowledge would help in the understanding of the properties of coffee by-products to facilitate their valorization and mitigate their negative effect on the environment.

## 2. Materials and Methods

Coffee (Arabica) husk, parchment and ground samples were kindly supplied by the Experimental Station in III Frente municipality, Santiago de Cuba, Cuba. All samples were collected from production batches of the 2021 harvest. Spent coffee ground samples were obtained in a traditional Italian coffee maker. Several replicates of some of the evaluated parameters were carried out for a better statistical analysis. Statistical comparison of the coffee residues with respect to each of the parameters was carried out through the Multiple Sample Test with 95% confidence and using the Statgraphics Centurion XVI.II software.

### 2.1. Field-Emission Scanning Microscopy (FESEM) and Energy-Dispersive X-Ray (EDX) Spectroscopy

FESEM (Zeiss AURIGA Compact, Jena, Germany) was used to observe the surface morphology with the carbon of the coffee by-products. The samples were coated with gold using a vacuum sputter coating machine (Leica EM ACE200, Wetzlar, Germany). Subsequently, they were placed in a 9-sample holder to be introduced into the microscope. The micrographs were taken at 1 KV. The structures were analyzed at a magnification of 100×.

### 2.2. Energy-Dispersive X-Ray (EDX) Spectroscopy

To study the elemental composition of the samples, EDX spectroscopy was performed using a scanning electron microscope Zeiss EVO MA10 with an EDX detector Oxford x-act.

### 2.3. Fourier Transform Infrared (FTIR) Spectroscopy

FTIR spectra were recorded using the attenuated total reflectance (ATR) mode Cary 630 spectrophotometer (Shimadzu Corporation, Duisburg, Germany, Europe). The three samples from Cuban coffee production were analyzed. FTIR spectral analysis was performed within the wave number range of 4000–500 cm^−1^ and 8 cm^−1^ resolution. The results were analyzed using OriginPro (2021 version, 9.8.0.200, OriginLab Corporation, Northampton, MA, USA).

### 2.4. Semi-Quantitative Analysis

The semi-quantitative composition of lignin, hemicellulose and cellulose in coffee husk, parchment and spent grounds was determined by the Chesson–Datta method [11]. To 1 g of dried sample (c), 150 mL of distilled water was added and the mixture was heated until boiling for 1 h in an oil bath. The mixture was filtered and the solid residue was washed with hot distilled water (300 mL). The residue was dried in an oven to constant weight (d). Then, it was mixed with 150 mL of H_2_SO_4_ 1 N and heated in an oil bath at 100 °C for 1 h. After that time, the suspension was filtered and washed with 300 mL of distilled water and the residue was dried (e). The dried solid was soaked in 10 mL of 72% H_2_SO_4_ at room temperature for 4 h. Afterward, 150 mL of 1 N H_2_SO_4_ was added into the mixture and refluxed for 1 h. The solid was washed with 400 mL of distilled water and dried in an oven at 105 °C to constant weight (f). Finally, the solid was burned at 650 °C in a furnace until it become ash, and was weighed (g). The percentage of hemicellulose, cellulose, lignin and ash was calculated as follows:

%hemicellulose = (e − d)/c × 100%(1)

%cellulose = (f − e)/c × 100%(2)

%lignin = (g − f)/c × 100%(3)

%ash = g/c × 100%(4)

### 2.5. Thermogravimetric Analysis (TGA)

The thermal stability of the different samples was determined by TGA measurements performed using a Mettler Toledo (Chile) thermogravimetric analyzer (TGA/SDTA 85-F). The amount of sample used for each measurement was ca. 1 mg. All measurements were performed under a nitrogen atmosphere with a gas flow of 10 mL min^−1^ by heating the material from room temperature to 600 °C at a heating rate of 10 °C min^−1^. The results were analyzed using Origin Program (2021 version, 9.8.0.200, OriginLab Corporation, Northampton, MA, USA).

### 2.6. Determination of Total and Insoluble Ashes (TAPPI T211 om-85)

In a previously tared and incinerated crucible, 2–3 g of dry and ground sample (h) was added. It was placed on the heating plate, then transferred to the furnace and the sample was gradually incinerated up to 650 °C. After obtaining white ashes, it was weighed every hour until constant weight (i). To the ashes, 25 mL of 2 mol/L hydrochloric acid was added, the crucibles were covered and placed on a heating plate for 5 min. The contents of the crucibles were filtered and washed with hot water until the washing waters were neutral. The insoluble ashes were dried and incinerated at 650 °C until constant weight (j). The percentage of total ashes and insoluble ashes was calculated as follows:

%Total Ashes = (h − i)/h × 100%(5)

%Insoluble Ash = (h − j)/h × 100%(6)

### 2.7. Determination of Relative and Apparent Density

In a 10 mL test tube containing 6 mL of water, 1 g of (i) was added and the displaced volume was observed. The difference between the original volume (j) and the displaced one (k) gives the volume of the sample, which was used as an input for Equation (7) to determine the relative density.

σ = i/(j − k)(7)

**Apparent density**. An empty test tube of 50 mL was weighted (m_0_). Next, the test tube was filled with the sample up to 50 mL capacity and weighted again (m_1_). The apparent density was obtained using Equation (8), where V is the volume of the tube.

σ_apparent_ = (m_1_ − m_0_)/V (8)

### 2.8. Determination of Extractive Content (TAPPI 264 om-88)

In Soxhlet equipment, 10 g of sample (a) was extracted with 400 mL of ethanol–benzene (1:3) for 6 h. Later, in the same Soxhlet, a second extraction of the sample was carried out with 400 mL of ethanol for 5 h and a third with 500 mL of distilled water for an hour. The sample was removed from the Soxhlet and washed with 500 mL of hot distilled water. Finally, the sample was allowed to dry in open air and weighed (b). The percentage of total extractives was calculated using Equation (8).

Total extractives (%) = (a − b)/a × 100%(9)

### 2.9. Phytochemical Screening

The ethanolic and aqueous extracts from coffee residues underwent qualitative phytochemical analysis [12]. The tests allowed for noticing the presence of various metabolites such as phenols, steroids, glycosides, saponins, flavonoids, terpenoids, alkaloids and reducing sugars.

### 2.10. pH Determination of Aqueous Extractives (TAPPI T252 om-90)

In a boiling flask, 10 g of sample was refluxed with 100 mL of distilled water. After 3 h, the solution was allowed to cool down to room temperature (~25 min) and the pH value was measured with a pH meter.

## 3. Results and Discussion

### 3.1. Field-Emission Scanning Microscopy (FESEM)

The morphology shows the interaction of the components in the materials and the effect of processing; ergo, this is a glimpse into the composition of the residue and its physico-chemical properties. Therefore, the SEM analysis is useful for quality control, ensuring that coffee waste meets certain standards before being used in specific applications, for example, the use of coffee biomass as an adsorbent, where changes in its morphology are observed by SEM after the adsorption process, thus evaluating the material’s capacity to be used for that purpose [13].

**Figure 2:**
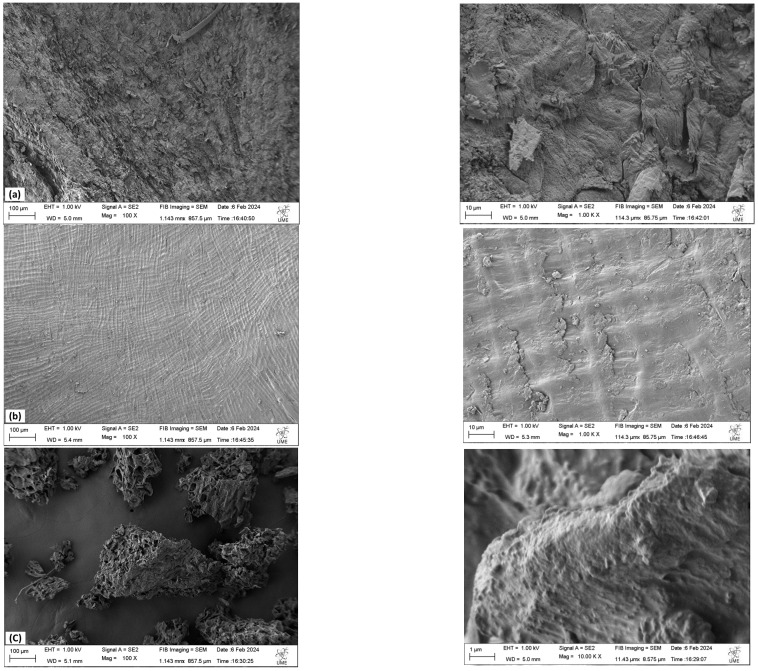
FESEM micrographies of the (**a**) coffee husk, (**b**) coffee parchment and (**c**) spent coffee grounds. On the left side, images of the three residues with a magnification of 100 μm are shown, whereas on the right side, images at the scale of 10 and 1 μm can be seen.

The morphologies of the residues were studied by analyzing the images obtained by FESEM. The FESEM images taken are shown in Figure 2. These images were taken with the deposition of an additional conductive layer and an electron acceleration (EHT) of 1 kV to decrease charge buildup at higher magnifications.

The images show that the three residues have a different and heterogeneous morphology. In the case of spent coffee grounds, it shows a porous structure like a sponge at a lower magnification, but at a higher magnification, the pores are very shallow and the structure seems fibrous. Poor mesoporosity and the absence of micropores in spent coffee ground has been previously reported by Ballesteros et al. [14] in 2014. On the other hand, an irregular, sheet-shaped surface is observed in the coffee parchment, with ridges along its surface, due to cellulose microfibrils [15]. In the coffee husk, a rough and cracked surface can be seen, with slight elevations, similar to that observed by Cruz and Crnkovic in 2015 [16]. Although they exhibit different shapes, all show a fibrous structure due to the presence of mainly cellulose in their composition. The variation in morphology across different reports may be in part due to climate variation that can wear down their surface and even due to the action of fertilizers or insects, but most importantly due to the processing previous to the analysis, like the grounding method, drying and extracting.

### 3.2. Energy-Dispersive X-Ray (EDX) Analysis

The analysis of the chemical composition of the samples was carried out by EDX analysis (Table 1). EDX analysis graphs appear in the Supplementary Material (Tables S2–S4).

In this case, the samples were analyzed with a carbon bridge touching the top surface of the samples. For a better determination of the chemical composition, X-ray spectroscopy was performed at various points on the surface of the samples. This allowed us to corroborate its heterogeneous composition, especially the percentage of minerals in them varied throughout the material. The EDX showed that C and O are present in high percentages in these residues. In the case of carbon, which is the element found in the greatest proportion, the percentages were as follows: 67.1% (coffee husk), 71.8% (coffee parchment) and 72.1% (coffee grounds). In addition to those mentioned, there was the presence of traces of K, Mg, P, S and Ca, with potassium being found at the highest percentage. Coffee husk is the one with the highest K content and is the only one that does not contain calcium, but Cl, Sn and In only appear in this residue. On the other hand, Mo and Cu are found exclusively in coffee parchment.

The results of the elemental composition analysis of the Cuban waste obtained were compared with previous reports. The coffee parchment composition reported by Arango-Agudelo et al. [17] has a high content of C (18%), Si (3.6%), K (37.1%), Cl (4.4%), Fe (4.3%) and Ca (20%). In the coffee grounds, only the presence of C (98.6%), Mg (0.3%), Ca (0.5%) and K (0.6%) was observed [14]. In coffee husks, Cruz and Crnkovic [16] reported that C (47%), O (48%) and K (4%) predominate, while elements such as Mg, Si, P, Cl, Ca and Fe were found in trace amounts.

One of the things that draws attention when comparing the results obtained by EDX with those reported in the literature is the variation in carbon content and that the element Si is not found in Cuban waste despite its signal appearing in the IR spectra. In the case of lignocellulosic materials, they have the peculiarity of being heterogeneous; that is, they will not have the same chemical composition throughout their surface. When using a localized technique, it will only provide elemental information from the sample region under analysis. This deficiency makes it difficult to identify and accurately quantify the individual elements in each material. In the case of carbon content, the high values obtained may be associated with the fact that they are lignocellulosic materials, which have a high content of this element. In addition, the carbon bridge that was added to the sample to improve the quality of the image and the analysis, could introduce an additional carbon signal into the spectrum, which could lead to an overestimation of the carbon content in the samples. It should be noted that one of the factors that can influence the variation in chemical composition is the variety of coffee grown in each region. In addition, growing conditions such as soil type, climate and agricultural practices also play an important role in the accumulation of carbon in coffee beans [17]. In the case of coffee husks, both come from the same variety, Arabica coffee, although one is of Cuban origin and the other Brazilian. In the study, it was observed by EDX that carbon and oxygen are the elements found in greater proportion, also highlighting the presence of potassium. These results are consistent with those obtained for Cuban coffee, although the proportions of these elements vary between the two samples.

As for the results reported by Arango-Agudelo et al. [17], it seems that this comes from a single EDX measurement on the residue, resulting on a more unreliable composition and explaining the significant differences with the present study.

### 3.3. Fourier Transform Infrared (FTIR) Spectroscopy

**Figure 3:**
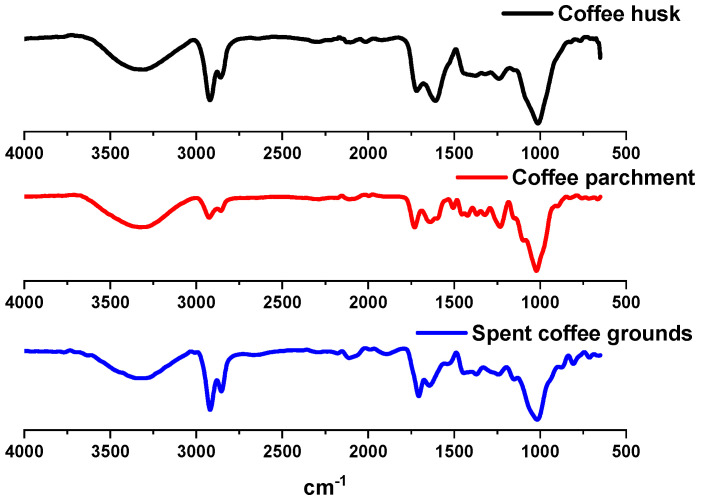
FTIR spectra of coffee husk (black), coffee parchment (red) and spent coffee grounds (blue). FTIR in the region between 4000 and 500 cm^−1^.

The analysis of the infrared spectrum offers information about the functional groups present in the residues. The FTIR spectra of the coffee waste are plotted in Figure 3. FTIR spectral analysis was performed within the wave number range of 500–4000 cm^−1^.

In this case, the three spectra are very similar because of the similar composition of lignocellulosic materials. These kinds of wastes are mainly composed by cellulose, hemicellulose and lignin [18]. Therefore, the most intense signals in the IR spectra will be associated with these compounds and the signals of the secondary metabolites present in these residues are overlapped with the signals of the above-mentioned biopolymers.

Table S1 of the Supplementary Material shows the assignments of the most intense signals and the compounds that may be associated; for this, we were guided by what was stated by Toribio-Cuaya et al. [19]. In all the spectra, intense signals associated with ν_-OH_, ν_Csp3−H_, ν_C=O_, ν_C=C_, γ _CH_ (ring deformation) and Si–O–Si (asymmetric stretching) can be seen, demonstrating the abundance of these groups in these wastes. In the case of the signal at 1236.07 cm^−3^ from the coffee parchment, it is more intense in relation to those of the other residues; this may be related to a greater abundance of syringyl alcohols in the lignin [20].

### 3.4. Semiquantitative Analysis

Analysis of the humidity, soluble compounds, hemicellulose, cellulose, lignin and ash was determined by the Chesson–Datta method (1981) [11]. This method does not have a high precision, but it is still useful to approximate the amount of the main components in waste and evaluate their suitability as possible raw materials. The basis of this test is the difference in the chemical properties and solubility of the main components of the material: lignin, cellulose and hemicellulose, which allows for their selective dissolution in acid of varying concentrations. The content of each component was calculated by measuring the relative mass loss of the solid waste in each step. This determination was carried out in triplicate. The resulting percentages and their standard deviations are shown in Table 2 and Table S5–S7.

The low moisture content in all three coffee byproducts can be attributed to the fact that coffee cherries must be dried to separate the husk and parchment from the coffee beans, and the beans are roasted before the preparation of the beverage. According to the obtained results, the husk, parchment and coffee grounds are composed mainly of cellulose, but also present a considerable amount of hemicellulose and lignin. The results obtained were compared with those reported in the literature (Table 3). Of note, the composition of the waste varies depending on the extraction method and the cultivation conditions.

The hemicellulose, cellulose and lignin contents in Cuban coffee wastes are in the range of what is to be expected from this kind of material, with cellulose being the main component. The obtained results are, for the most part, in accordance with the values reported in the scientific literature. Table 3 shows scientific reports on the composition of cellulose, hemicellulose and lignin in coffee waste. The low ash contents obtained are due to the treatments for the extraction of the components during the analysis. The main compounds in coffee waste ash are silica and other inorganic salts. The silica in the biomass can dissolve in concentrated acid [28], so, during the Chesson–Datta method, the silica that might be present in coffee waste was extracted, causing a possible overestimation of hemicellulose and cellulose content. This is verified by the determination of total ash in this work.

It is important to note that the composition of coffee byproducts heavily depends on the kind of coffee, the conditions during its growth, the region of cultivation and the processing after harvest. Also, the method used in the content determination is important to get reliable results. As stated before, the Chesson–Datta method is only approximated and suitable for semiquantitative analysis.

The biopolymers present in the waste studied are of vital importance because they can be used to produce additives in cosmetics, food and biomaterials; improve soil quality; and produce paper and pharmaceutical products, among others [18]. On top of these potential uses, the high content of these polymers in the by-products could have a significant impact on their use within the biofuel industry. These lignocellulosic materials can be beneficial, as they provide a great amount of raw material for the production of biofuels, but complex composition makes their decomposition more difficult, which may require more intensive chemical or enzymatic treatments, increasing the costs and complexity of the process [18]. However, the interactions between constituents and the catalytic influence of the minerals naturally present in biomass can modify significantly the yields of some products [18].

### 3.5. Thermogravimetric Analysis (TGA)

Thermal analysis is the main method of characterizing the thermal properties of a material. The pyrolytic decomposition of lignocellulosic materials in an inert atmosphere has been widely studied [29].

**Figure 4:**
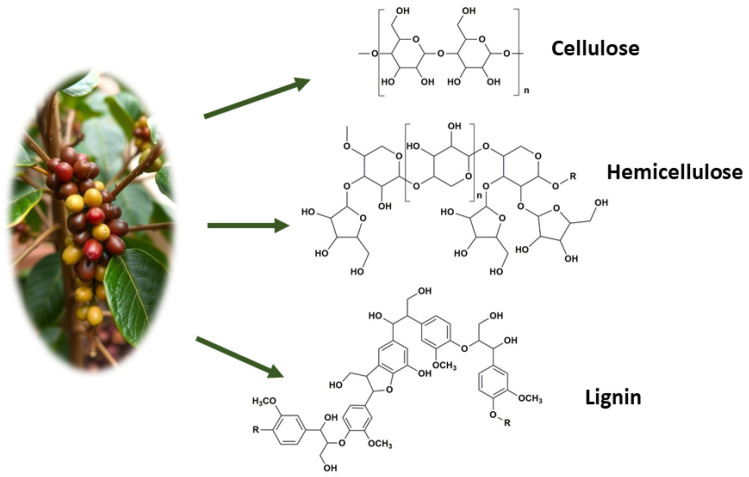
Schematic representation of cellulose, hemicellulose and lignin fibers, and the structures of the plant cell wall.

The thermal decomposition of biomass proceeds via a very complex set of competitive and concurrent reactions due to the mixture of polymers in its composition. The DTGA curves of lignocellulosic materials can be approximately considered as a sum of the DTGA curves for its isolated main components, which are hemicellulose, cellulose and lignin. Figure 4 shows the chemical restructure of these polymers.

The degradation of hemicellulose, the least thermally stable lignocellulosic component [29] takes place in the range of 200–300 °C. This biopolymer is composed of various saccharides (xylose, mannose, glucose, galactose, etc.) that form an amorphous structure, highly branched, which is easy to remove from the fiber and degrade thermally in the form of volatile releasing gases (in particular, CO, O_2_ and some hydrocarbons) at relatively low temperatures.

Cellulose is a long polymer that has linear glucose chains, with an ordered and highly crystalline structure, forming a very strong material with high thermal stability. The thermal decomposition of cellulose takes place in two major steps: depolymerization of residual cellulose followed by formations of glycosans [30]. The decomposition of cellulose shows in the DTGA as an abrupt mass loss with a small temperature interval, usually between 300 and 400 °C.

Finally, lignin is the most difficult to degrade under these conditions [15]. Lignin is mainly composed of phenylpropanoid units, forming a highly crosslinked and branched network. Lignin decomposition takes place slower than other components and over a larger temperature interval, usually between 200 and 600 °C. The decomposition of lignin takes place in two stages. Between 200 and 400 °C, primary pyrolysis reactions occur, with a DTG peak around 350 °C, and above 400 °C, secondary pyrolysis reactions take place.

Due to the concurrent reactions taking place over the same temperature range, DTGA peaks can be superimposed and will predominate the behavior of the main component. The cellulosic contribution predominates in the thermal behavior lignocellulosic materials, mainly due to the larger proportion of cellulose and its higher reactivity.

**Figure 5:**
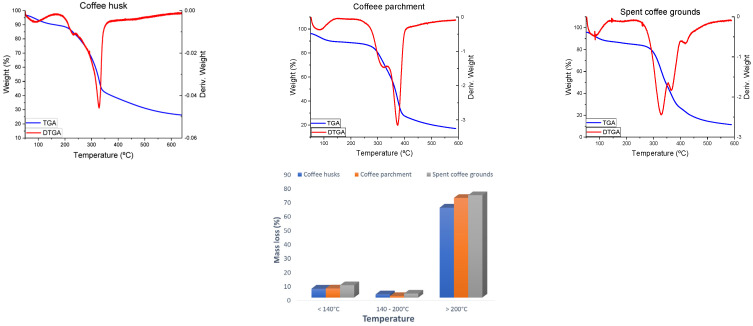
TGA results of coffee husk, coffee parchment and spent coffee grounds, and mass loss (%) and temperature range at which maximum decomposition occurs.

Figure 5 (Table 4) shows the thermo-gravimetric weight loss curve (TGA) and the differential curve (DTGA) corresponding to the three lignocellulosic wastes. Table 4 shows mass losses (%) in the TGA of coffee residues. The first zone is below 200 °C, the second zone between 200 and 400 °C, and the third zone above 400 °C. In the first zone, the release of adsorbed moisture and other volatile compounds takes place. All three wastes show a peak below 150 °C with a mass loss between 6% for coffee husk and 9% for spent coffee grounds.

The second zone in the thermogram corresponds to active pyrolysis, where the main mass loss takes place. The hemicellulose decomposition occurs first and can be seen in all three thermograms as peaks around 330 °C. The hemicellulose peaks form shoulders with the peaks corresponding to the cellulose decomposition, which takes place above 350 °C. The intensity of the peaks depends on the composition of each waste. The larger peak centered on 374 °C in the DTGA of coffee parchment corresponds to a higher cellulose content compared to the other coffee wastes, while coffee husks and spent coffee grounds show larger peaks corresponding to hemicellulose decomposition at a lower temperature, due to a higher hemicellulose content. The higher hemicellulose content in coffee husk could explain the lower main decomposition temperature of this material. The DTGA peaks above 350 °C also coincide with the primary pyrolysis reactions of lignin.

In the third zone of the thermogram, passive pyrolysis occurs, where the decomposition of lignin remnants via secondary pyrolysis reactions takes place. This slower decomposition can be seen as a tailing in the thermogram of coffee husk and coffee parchment. In the spent coffee ground DTGA, a peak centered around 418 °C can be observed due to a higher lignin content in the sample.

The obtained thermograms are similar to the previously reported thermal behavior of coffee wastes, although with some differences due to coffee varieties and place of cultivation. As reported by Bongomin et al. [31], for coffee husk, cellulose decomposition takes place at 345 °C and the lignin degradation peak is at 486 °C.

The TGA curve obtained by Reis et al. [15] indicates that Brazilian coffee parchment has a different behavior than that obtained, since its maximum thermal decomposition corresponding to cellulose is at 330 °C. In the case of Cuban parchment, its maximum is found at a higher temperature, coinciding with the primary decomposition of lignin. The curve obtained for spent coffee grounds is similar to that of Wachter et al. [32] in 2022 in an argon atmosphere, whereby it was observed that the maximum decomposition appears at 337 °C.

The slight variation in decomposition temperatures relative to the ones reported in the literature can be explained by the location and conditions of cultivation, the processing and the previous treatments to which these biomasses were subjected, influencing the amount of cellulose and lignin present in the material, and their thermal properties.

### 3.6. Determination of Total and Insoluble Ashes

The determination of total and insoluble ashes was carried out as described in the previous section. The results are summarized in Table 5 and Table S8.

Coffee husk is the waste that has the highest amount of total ash and one of the lowest amounts of insoluble ash. In all cases, the results obtained were within the range reported in the literature [8,22,23,33]. These results prove that the main components of ash in coffee waste are soluble in acid, making its determination via the Chesson–Datta method impossible.

### 3.7. Determination of Relative and Apparent Density

The determinations of relative and apparent densities were carried out as described in the previous section. The results are summarized in Table 6 and Table S8.

The described tests are governed by different standards for the study of charcoal due to plant fibers behaving similar to charcoal when they are incinerated [37]. Many materials made from agricultural waste require that they have a low or medium density. These can be used to obtain construction materials, clays or as an additive. In many coffee wastes, their ability to retain water is also measured through apparent density. It is said that they can be part of clays, perlites, vermiculites, expanded polystyrenes, natural corks or cellular glass, as long as they are light loads since they have an apparent density of less than 800 kg/cm^3^ [38]. The relative densities of the coffee husk and grounds are quite close and are higher than that of the coffee parchment. When comparing these values with those reported in the literature, the coffee husk and parchment have values similar to those reported [34,35], while the Cuban coffee grounds have a higher value than that reported by Ciesielczuk et al. [36]. This result may be due to several factors. First, the roasting of the beans influences their chemical composition and the amount of oils and soluble compounds that are released during brewing. A darker roast can result in a denser ground due to the greater amount of oils that are extracted [36]. Brewing techniques also affect the density of the biomass, as methods that allow for a greater extraction of compounds such as oils tend to produce denser grounds. Cuban coffee was obtained from a Moka pot, which tends to extract more oils, and therefore the coffee grounds would be denser.

### 3.8. Determination of Extractive Content, Phytochemical Screening and pH Determination of Aqueous Extractives

To determine the percentage of secondary metabolites contained in the residues and qualitatively identify the different types of compounds they contain, experiments of extractive content and phytochemical screening were carried out. In addition, the pH of the waste was determined. The obtained results are shown in Table 7.

To determine the percentage of secondary metabolites present in coffee waste, their extractive content was determined. As seen in Table 7 and Table S8, the highest percentage of extractive content was presented in coffee husk (30.9%), followed by grounds at 27.3%, and finally the parchment at only 6.9%. In the case of coffee husk, the value of extractive content was higher than that reported by Van Nguyen et al. [39] (9%) and Berhanu et al. [40] (8%). In the case of Van Nguyen, he exclusively refluxed the plant material in acetone for extraction, while Berhanu performed the extraction in three steps and using Soxhlet equipment. The first step was a mixture of toluene and ethanol (2:1). In the second step, they used ethanol, and in the third, the solvent was water. Although the last author’s extraction technique was similar to the one carried out in this report, the extractive content of the Cuban coffee husk was much higher than those reported. This result may be influenced by the polarity of the solvents used. In our case, we used benzene, which is more nonpolar than toluene, and we also used it in a greater proportion; therefore, a greater number of nonpolar compounds could be extracted. It should be noted that in both the coffee husk and the coffee grounds, there is a high percentage of water-soluble components (39% and 23%, respectively), which were determined by the Chesson–Datta method using gravimetry. This high percentage of soluble components also influences the result of the extractives content, hence its high value when compared to those reported. On the other hand, the result obtained for coffee grounds was similar to that obtained by Andrés et al. [41] applying the TAPPI t 5 os-73 Standard, which reported 30.6% extractives using ethanol and benzene as solvents. In the case of coffee parchment, although the extraction yield was the lowest, this value was higher than those obtained by other authors [42,43]. Rosa A. and Juan D. [43] report an extraction yield of 0.44% using the same extraction technique, while Ana M. [42] reported 3.09% using acetone, methanol and water acidified with 2N HCl in the extraction. In this case, Cuban coffee parchment contained a greater number of secondary metabolites than those reported in the literature; this result can be attributed to a combination of environmental, genetic, and agronomic management and/or coffee processing factors.

As part of the characterization of these wastes, phytochemical screening was carried out. This method allowed us to qualitatively determine the main groups of the chemical compounds present in the waste, which could have been extracted using the extractive content method. This could guide the fractionation of the extracts for the isolation of compounds or groups of compounds of greatest interest for a given purpose. It is known in advance that coffee waste contains bioactive components, which if recovered would be potentially useful for the pharmaceutical and food industries [44]. For this study, two extracts from different solvents were used, as shown in Table 7. Using this method, it can be observed that the waste has a diversity of compounds, of which the concentration varies depending on the solvent with which they are extracted. Of all the compound classes that can be determined by the screening, those that gave a positive reaction in the extracts evaluated were alkaloids, flavonoids, tannins, triterpenes and steroids, quinones and reducing sugars. These results are in correspondence with those reported in Table 8, with coffee grounds being the one that present the greatest evidence of the chemical groups detected in the screening, that is, a greater variety in compounds. The positive results of the phytochemical screening of coffee waste highlight its potential as a beneficial source of compounds with varied biological activity, which promotes a more sustainable obtention approach. Among the groups of compounds that abound in these wastes were alkaloids, which are widely used as psychotropic drugs, for neurodegenerative diseases and diabetes control, among others. Other groups of compounds that abound in these wastes are flavonoids, which have antioxidant, anticancer, anti-inflammatory and antibacterial properties. Triterpenes and steroids are also abundant, which can inhibit tumor cell lines in the prostate, breast and leukemia, and reduce liver inflammation [45].

In the screening, the presence of alkaloids in fractions C1, C2 and D can be highlighted. In the case of fraction C1, which is an acid fraction, the alkaloids present in the extracts are in salt form, making it difficult for them to react with the Mayer, Hager, Dragendorff and Wagner reagents. Being an aqueous solution, there may be other nitrogen compounds in the extracts such as amino acids and proteins [45] that react with the reagents and generate a false positive. For fractions C2 and D, the extracts were alkaline. This causes the deprotonation of the nitrogen present in the alkaloids, allowing for their extraction with organic solvents and their identification. The presence or absence of this type of secondary metabolite in the extracts was confirmed in fractions C2 and D. In the case of fraction C2, the extraction was in chloroform, and low-polarity alkaloids were identified, while in fraction D, medium-polarity alkaloids were found and the extraction was chloroform: ethanol (3:2). As can be seen in Table 7, low-polarity alkaloids were found in the ethanolic extract, while medium-polarity alkaloids appeared in the aqueous extract. In the case of coffee grounds, since it was the residue obtained from the extraction of soluble compounds from ground coffee using hot water, the alkaloids that could be present in the aqueous extract had already been extracted when obtaining the beverage. In this residue, only the presence of alkaloids in the ethanolic extract, both of medium and low polarity, appeared, with low-polarity alkaloids being more abundant. Scientific reports have shown that some of the most abundant alkaloids in coffee grounds are caffeine, theobromine, theophylline and trigonelline [45]. Metabolites such as triterpenes and steroids were found mostly in the ethanolic extract with a yellow color, while in the aqueous extract, they only appeared in fraction D of the coffee grounds. This assay was violet in color, demonstrating the presence of low-polarity triterpenes. It is also observed that there is a greater proportion of flavonoids in fraction E; the positive result of this test was probably due to the presence of high-polarity glycosylated flavonoids. The amyl phase of the test in most cases presents an orange color indicating the presence of flavones or flavonols. In our case, the aqueous extracts of parchment and coffee grounds stood out for presenting a greater abundance of them. In fraction E of the aqueous extract, the presence of catechins and proanthocyanidins was identified; the presence of these compounds is in accordance with what has been reported in the literature [45]. The waste that presents the greatest abundance of them was coffee grounds.

In the case of pH, it is important to know the value at which the different residues fluctuate. Through this parameter, you can control the chemical reactions in the manufacturing process of some types of materials or allow for compatibility with other pH-sensitive materials. Table 7 shows the results of the determinations and Table 8 shows the values exposed in the literature of these wastes. Regarding pH, the three residues had lower values than those in the literature. The highest pH was found in parchment (4.91) followed by lint (4.87). In all cases, the order of basicity reported in the literature for these three wastes was maintained. The decrease in the pH of these residues compared to those reported in the literature may be due to a greater generation of metabolites such as organic acids (such as chlorogenic acid and caffeic acid), those formed from carbohydrate degradation products that are fermented and produce organic acids (such as lactic acid and acetic acid) or due to the presence of phenolic compounds (such as flavonoids and tannins).

## 4. Conclusions

The generation of waste or by-products during coffee production has both negative and positive impacts depending on the point of view from which it is observed. They can generate pollution due to their inadequate final disposal or they can become agents that improve the health of the environment. In this article, three Cuban *Arabica* coffee residues (husk, parchment and coffee grounds) were characterized, demonstrating that they have a high potential for use thanks to their varied chemical and physicochemical composition. The presence of minerals in their chemical composition determined by EDX allows us to use them in agricultural practices as fertilizer, with coffee grounds standing out for having more minerals than reported in the literature. Various techniques were used to corroborate and semiquantify the presence of cellulose, hemicellulose, lignin and silica in the residues (TGA, IR, Chesson–Datta method). Using EDX, it was observed that the biomasses were mainly composed of carbon (C) and oxygen (O), as expected for natural biopolymers. In addition, using the Chesson–Datta method, it was confirmed that these biopolymers were present in a higher proportion relative to the secondary metabolites determined as the extractive content. The density and pH of the waste were determined. The coffee husk and parchment presented relative density values similar to those reported in the literature, while the coffee grounds presented a value higher than that reported, but similar to the other two biomasses. This result may be related to the loss of compounds such as oils during both roasting and coffee preparation. In the case of pH, it ranged between 4.25 and 4.91, presenting values lower than those in the literature. And finally, the extractive content was determined and phytochemical screening was carried out to determine the main groups of secondary metabolites that these coffee residues present. The highest percentage of extractive content was found in the coffee husk (30.9%), followed by the grounds at 27.3%, and finally, there was the parchment at 6.9%. It should be noted that both the coffee husk and coffee grounds had a high percentage of water-soluble components (determined by the Chesson–Datta method) that may influence the result of the extractive content. In the case of parchment, although the extraction yield was the lowest, this value was higher than those obtained by other authors. The results of the phytochemical screening are in accordance with what has been reported, with the coffee grounds presenting the greatest variety in compounds. Furthermore, statistical results applying the multiple-sample test demonstrated with 95% confidence that coffee residues present varied properties. Overall, these results demonstrate that the characteristics of coffee residues depend on the type of residue, the location of cultivation and the conditions under which they are grown.

## Figures and Tables

**Table 1:** EDX analysis of the coffee husk, coffee parchment and spent coffee grounds.

Element	Sample		
	Coffee Husk	Coffee Parchment	Spent Coffee Grounds
**C**	67.1 ± 1.6 ^A^	71.8 ± 5.5 ^B^	72.1 ± 5.2 ^B^
**O**	25.8 ± 1.3 ^A^	25.0 ± 2.4 ^A^	24.3 ± 1.4 ^A^
**Mg**	0.2 ± 0.1 ^A^	0.22 ± 0.04 ^A^	0.3 ± 0.2 ^A^
**S**	0.11 ± 0.04 ^A^	0.14 ± 0.05 ^A^	0.2 ± 0.2 ^A^
**K**	5.5 ± 1.0 ^A^	0.52 ± 0.04 ^B^	2.3 ± 2.5 ^C^
**P**	0.20 ± 0.04	-	0.4 ± 0.6
**Cl**	0.7 ± 0.1	-	-
**Sn**	0.7 ± 0.1	-	-
**In**	0.5	-	-
**Ca**	-	2.2 ± 4.2	0.7 ± 0.9
**Mo**	-	0.27	-
**Cu**	-	0.24	-

(^A, B and C^) equal letters indicate that there are no significant differences with 95% confidence by the multiple data comparison test between the residuals.

**Table 2:** Semi-quantitative analysis of Cuban coffee waste.

Material	Humidity (%)	Soluble Compounds (%)	Hemicellulose (%)	Cellulose (%)	Lignin (%)	Ash (%)
**Coffee husks**	8.8 ± 0.4 ^A^	39.0 ± 0.7 ^A^	12.3 ± 0.8 ^A^	31 ± 1 ^A^	16 ± 1 ^A^	0.04 ± 0.03 ^A^
**Coffee parchment**	8.4 ± 0.6 ^A^	12 ± 1 ^B^	13.0 ± 0.8 ^A^	51 ± 4 ^B^	21 ± 1 ^B^	0.4 ± 0.1 ^B^
**Spent coffee grounds**	6.9 ± 0.8 ^B^	23.1 ± 0.9 ^C^	22.6 ± 0.7 ^B^	29.7 ± 0.8 ^A^	22.7 ± 0.9 ^B^	0.06 ± 0.03 ^A^

(^A, B and C^) equal letters indicate that there are no significant differences with 95% confidence by the multiple-data comparison test between the residuals.

**Table 3:** Scientific reports on the composition of cellulose, hemicellulose and lignin of coffee waste.

	Waste		
Compound	Coffee Husk	Coffee Parchment	Spent Coffee Grounds
**Ashes**	3–10% [21]	0.38–1% [21,22]	0.43–2.20% [23]
**Cellulose**	14.7–57.1% [24]	40–49% [15,25]	8.6–24.3% [26]
**Hemicellulose**	10.2–29.7% [24]	25–32% [15,24]	24.8–36.7% [26]
**Lignin**	10.1–34.2% [24]	33–35% [15,24]	23.9–33.6% [14,27]

**Table 4:** Mass loss (%) and the temperature at which maximum decomposition occurs in the TGA of coffee residues.

Residue	<140 °C	140–200 °C	>200 °C	Total Mass Loss (%)
**Coffee husk**	6.36	2.29 (153 °C)	64.35 (231 °C, 327 °C)	73.00
**Coffee parchment**	6.54 (84 °C)	1.10 (197 °C)	71.28 (324 °C, 374 °C)	78.91
**Spent coffee grounds**	8.81 (84 °C)	2.80 (185 °C)	73.24 (329 °C, 366 °C, 418 °C)	84.85

**Table 5:** Content (%) of total and insoluble ashes.

Test	Sample		
	Coffee Husk	Coffee Parchment	Spent Coffee Grounds
**Total ashes (%)**	6.41 ± 0.08 ^A^	1.0 ± 0.2 ^B^	2.2 ± 0.1 ^C^
**Insoluble ashes (%)**	0.050 ± 0.003 ^A^	0.24 ± 0.02 ^B^	0.076 ± 0.005 ^C^

(^A, B and C^) equal letters indicate that there are no significant differences with 95% confidence by the multiple data comparison test between the residuals.

**Table 6:** Relative and apparent density of coffee waste.

Test	Sample		
	Coffee Husk	Coffee Parchment	Spent Coffee Grounds
**Relative density (kg/m^3^)**	579 ± 13 ^A^	321 ± 4 ^B^	514 ± 8 ^C^
**Apparent density (kg/m^3^)**	198 ± 5 ^A^	269 ± 21 ^B^	425 ± 18 ^C^
**Relative density reported in the literature (kg/m^3^)**	550–850 [34]	323 [35]	313.1 [36]

(^A, B and C^) equal letters indicate that there are no significant differences with 95% confidence by the multiple data comparison test between the residuals.

**Table 7:** Phytochemical screening, total extractives and pH of the ethanolic and aqueous extract of three coffee residues.

Compound		Sample					
		Coffee Husk		Coffee Parchment		Spent Coffee Grounds	
		Ethanol	Water	Ethanol	Water	Ethanol	Water
**Fraction A**	**Tannins**	+	+	+	+	+++	+
	**Phenols**	-	-	-	+	-	+
**Fraction B**	**Triterene/steroid**	+++	-	+++	-	+++	-
	**Quinon**	+++	-	+	-	+++	-
**Fraction C1**	**Alkaloid**	+	+	++	+	+++	+++
**Fraction C2**	**Alkaloid**	++	-	++	-	++	-
	**Triterenes/steroid**	+	-	+	-	+	-
**Fraction D**	**Flavonoid**	-	-	-	-	+	-
	**Alkaloid**	-	++	-	++	+	-
	**Triterenes/steroid**	-	-	+	-	++	+++
**Fraction E**	**Reducing sugar**	+++	+	+++	+	+++	+
	**Proanthocyanidin/catechin**	-	+	-	++	-	+++
	**Flavonoid**	+	+	+	+++	++	+++
**Total extractives (%)**		30.9 ± 1.5 ^A^		6.9 ± 0.7 ^B^		27.3 ± 1.4 ^C^	
**pH**		4.25 ± 0.04 ^A^		4.91 ± 0.03 ^B^		4.87 ± 0.03 ^B^	

Precipitate or coloration: very abundant, +++; abundant, ++; middle, +; not detected, - (the number of positive signs indicated the observed color intensity after the reactions). (^A, B and C^) equal letters indicate that there are no significant differences with 95% confidence by the multiple data comparison test between the residuals.

**Table 8:** Percentage of secondary metabolites from coffee residues and pH reported in the literature.

Compound	Waste		
	Coffee Husk [21,46]	Coffee Parchment [21,35]	Spent Coffee Grounds [36,42]
**Proteins**	4–12%	0.4–2%	9.8%
**Carbohydrates**	16–89%	0.45%	34–39%
**Lipids**	0.5–3%	0.6%	16.2%
**Tannins**	1.8–9.3%	-	0.78–5.60%
**Reducing sugar**	14%	-	0.10%
**pH**	5.98	7.49	6.08

## Data Availability

The original contributions presented in the study are included in the article/Supplementary Material, further inquiries can be directed to the corresponding authors.

## Supplementary Materials

The following supporting information can be downloaded at: https://www.mdpi.com/article/10.3390/foods13213348/s1, Table S1: Assignments in the FT-IR spectra of coffee residues; Table S2: EDX analysis of the coffee husk; Table S3: EDX analysis of the coffee parchment; Table S4: EDX analysis of the spent coffee grounds; Table S5: Semiquantitative analysis of coffee husk; Table S6: Semiquantitative analysis of coffee parchment; Table S7: Semiquantitative analysis of spent coffee grounds; Table S8: Determination of total and insoluble ashes, relative density, apparent density, determination pH and total extractives of coffee residues; Figure S1: Phytochemical screening.
