# Supplementary material for: Preliminary Physical and Chemical Characterization of By-Products from Cuban Coffee Production
Source: Foods. 2024 Oct 22;13(21):3348. doi: 10.3390/foods13213348 (PMC11545320; doi:10.3390/foods13213348)
Supplement: Supplementary file 1 [file foods-13-03348-s001.zip › foods-3224530-supplementary.pdf]

foods

Article

Preliminary Physical and Chemical Characterization of By-Products from Cuban Coffee Production

Dayana Mesa [1], Juan P. Figueroa [1], Eduardo A. Leyes [1], Carlos R. Castillo [1], Amanda Collazo [1], Harold A Núñez [1], Dayamí Viltres [2], Yaneris Mirabal [3,*] and Yamilet Coll [1,*]

[1] Center for Natural Product Research, Faculty of Chemistry, University of Havana, Zapata and G, Havana 10400, Cuba; dayana.mesa@fq.uh.cu (D.M.); juan.figueroa-macias@mtl.maxplackschools.de (J.P.F.); eduardoleyes73@gmail.com (E.A.L.); crcastillossp@gmail.com (C.R.C.); acollazoaldana@gmail.com (A.C.); agusto71@gmail.com (H.A.N.)
[2] Instituto de Investigaciones Agroforestales Cruce de los Baños, Santiago de Cuba 92700, Cuba; dayamibarbanviltres@gmail.com
[3] Faculty of Engineering, Institute of Applied Chemistry, Universidad Autónoma de Chile, Talca 3460000, Chile
* Correspondence: yaneris.mirabal01@uautónoma.cl (Y.M.); yamcoll@fq.uh.cu (Y.C.)

Supplementary Materials: Table S1: Assignments in the FT-IR spectra of coffee residues; Table S2: EDX analysis of the coffee husk; Table S3: EDX analysis of the coffee parchment; Table S4: EDX analysis of the spent coffee grounds; Table S5: Semiquantitative analysis of coffee husk; Table S6: Semiquantitative analysis of coffee parchment; Table S7: Semiquantitative analysis of spent coffee grounds; Table S8: Determination of total and insoluble ashes, relative density, apparent density, determination pH and total extractives of coffee residues; Figure S1: Phytochemical screening

Table S1: Assignments in the FT-IR spectra of coffee residues.

Assignation	Coffee husks	Coffee parchment	Spent coffee grounds	Associated compounds
ν_{OH}	3316.9	3315	3317.7	Cellulose, Hemicellulose Lignin
ν_{C-H} symmetric; in -CH3 and -CH2	2915.5	2933.7	2918.9	Cellulose, Lignin
ν_{C-H} asymmetric; in -CH3 and -CH2	2858.6	2854.6	2856.7	Cellulose, Lignin
$\nu_{C=O}$ Carboxylic acid	1718.5	1731.2	1706	Lignin
δ_{OH}, adsorbed water<br>$\nu_{C=O}$ Carbonyl groups in conjugated p-substituted aryl ketones	1619.1	1638.3	1647.2	Cellulose<br>Lignin
Characteristic of aromatic rings due to the aromatic skeletal vibrations	-	1511.2	1524.1	Lignin
$\delta_{-CH2-CO-}$	1442.9	1452.2	1452.2	Hemicellulose
Aliphatic C-H in CH3 not in OMe	1380.6	1366	1368.8	Lignin
Syringyl ring (4) breathing with ν_{C-O}	1323	1325	-	Lignin
Syringyl ring (4) breathing	1240.4	1236.1	1244.3	Lignin
Aromatic C-H in plane deformation of syringyl type (4)	1145.6<br>↓	1146.5	1155.4	Lignin
*Aromatic C-H in plane deformation guaiacyl type (5) and C-O deformation of primary alcohol<br>*C-OH deformation of primary alcohol<br>*Si–O–Si asymmetric stretching	1013.9	1025.4	1013.1	Lignin<br>Cellulose<br>Silice

Table S2: EDX analysis of the coffee husk

CH site 1

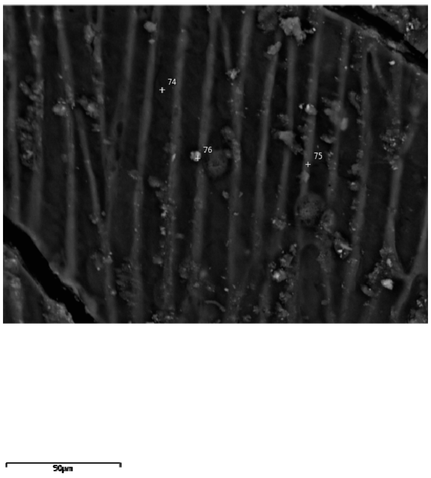

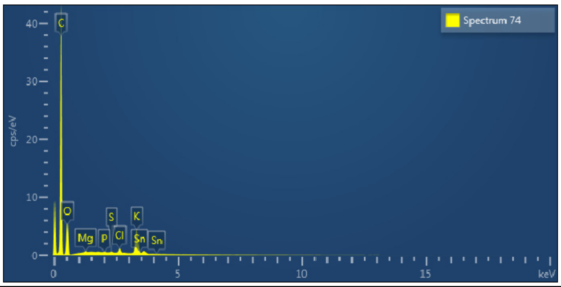

Element	Wt%	Wt% Sigma	Atomic %
**C**	66.52	0.32	74.87
**O**	27.32	0.31	23.08
**Mg**	0.13	0.02	0.07
**P**	0.12	0.02	0.05
**S**	0.12	0.02	0.05
**Cl**	0.60	0.02	0.23
**K**	4.54	0.06	1.57
**Sn**	0.64	0.09	0.07
**Total:**	100.00		100.00

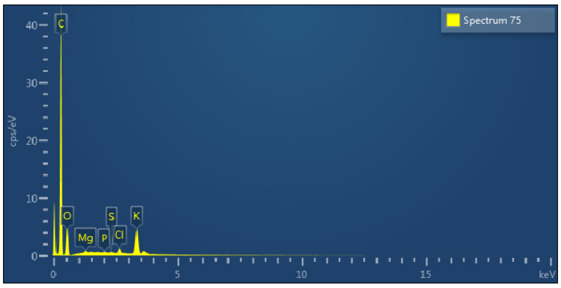

Element	Wt%	Wt% Sigma	Atomic %
**C**	68.98	0.31	76.84
**O**	25.22	0.31	21.09
**Mg**	0.18	0.03	0.10
**P**	0.11	0.02	0.05
**S**	0.11	0.02	0.05
**Cl**	0.74	0.03	0.28
**K**	4.67	0.06	1.60
**Total:**	100.00		100.00

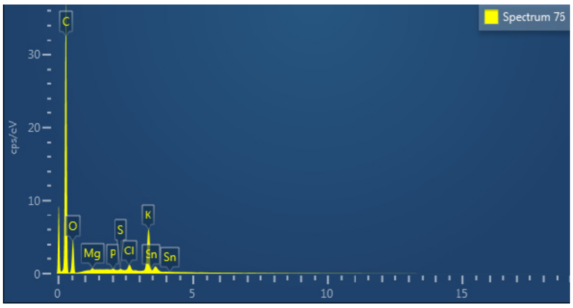

Element	Wt%	Wt% Sigma	Atomic %
C	64.61	0.33	74.16
O	26.42	0.32	22.76
Mg	0.20	0.03	0.12
P	0.12	0.02	0.05
S	0.16	0.02	0.07
Cl	0.66	0.03	0.26
K	7.08	0.08	2.49
Sn	0.75	0.11	0.09

Total:	100.00		100.00

CH site 2

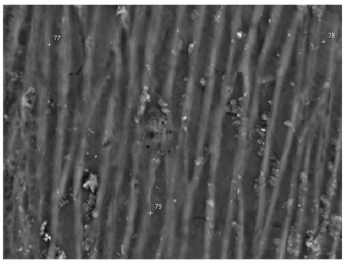

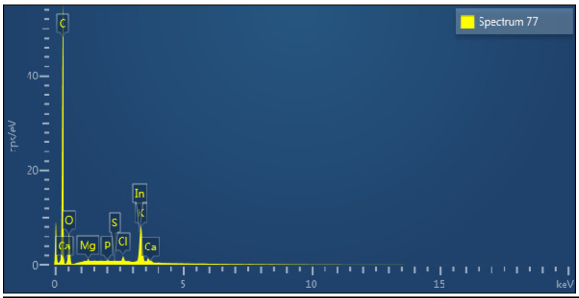

Element	Wt%	Wt% Sigma	Atomic %
**C**	66.53	0.34	75.56
**O**	25.34	0.33	21.61
**Mg**	0.27	0.03	0.15
**P**	0.15	0.02	0.07
**S**	0.14	0.02	0.06
**Cl**	0.78	0.03	0.30
**K**	5.93	0.08	2.07
**Ca**	0.38	0.03	0.13
**In**	0.48	0.13	0.06
**Total:**	100.00		100.00

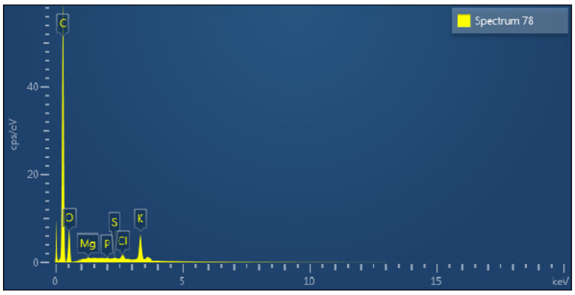

Element	Wt%	Wt% Sigma	Atomic %
**C**	67.25	0.32	75.35
**O**	26.76	0.31	22.51
**Mg**	0.19	0.03	0.10
**P**	0.08	0.02	0.04
**S**	0.12	0.02	0.05
**Cl**	0.66	0.03	0.25
**K**	4.94	0.06	1.70
**Total:**	100.00		100.00

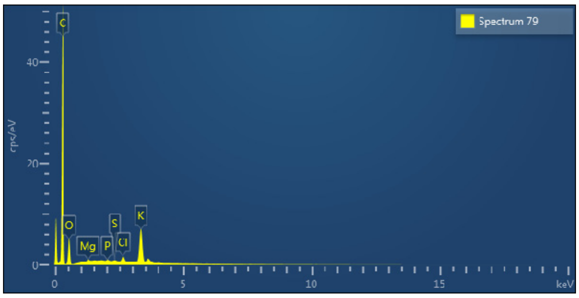

Element	Wt%	Wt% Sigma	Atomic %
**C**	68.94	0.33	77.39
**O**	23.73	0.32	19.99
**Mg**	0.21	0.03	0.12
**P**	0.16	0.02	0.07
**S**	0.09	0.02	0.04
**Cl**	0.77	0.03	0.29
**K**	6.10	0.07	2.10
**Total:**	100.00		100.00

Table S3: EDX analysis of the coffee parchmen

CP site 1

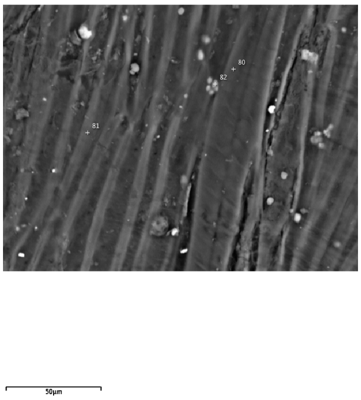

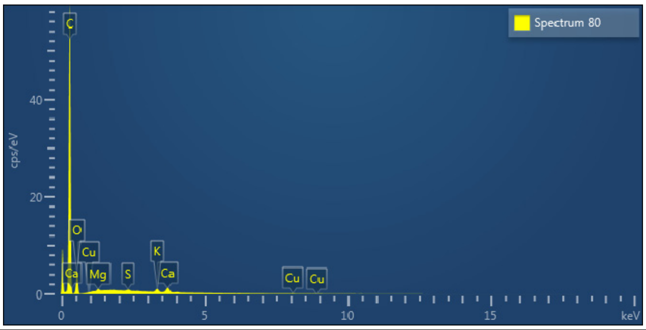

Element	Wt%	Wt% Sigma	Atomic %
C	75.59	0.31	81.36
O	22.11	0.31	17.86
Mg	0.24	0.03	0.13
S	0.15	0.02	0.06
K	0.63	0.03	0.21
Ca	1.03	0.04	0.33
Cu	0.24	0.07	0.05
Total:	100.00		100.00

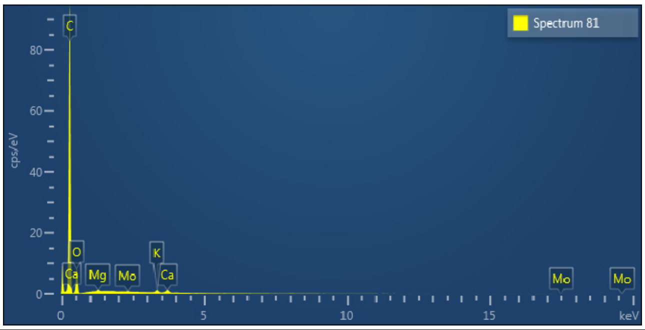

Element	Wt%	Wt% Sigma	Atomic %
C	74.21	0.30	79.91
O	24.24	0.29	19.60
Mg	0.19	0.02	0.10
K	0.48	0.02	0.16
Ca	0.60	0.03	0.19
Mo	0.27	0.06	0.04
Total:	100.00		100.00

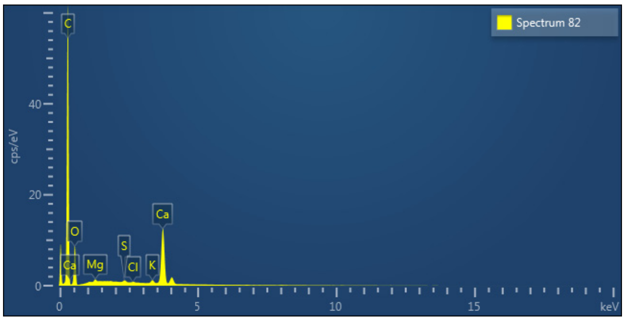

Element	Wt%	Wt% Sigma	Atomic %
C	61.02	0.32	71.13
O	28.86	0.32	25.26
Mg	0.24	0.03	0.14
S	0.18	0.02	0.08
Cl	0.09	0.02	0.03
K	0.46	0.03	0.16
Ca	9.15	0.09	3.20
Total:	100.00		100.00

CP site 2

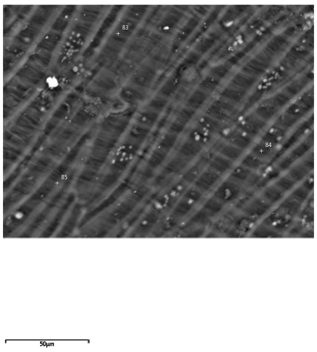

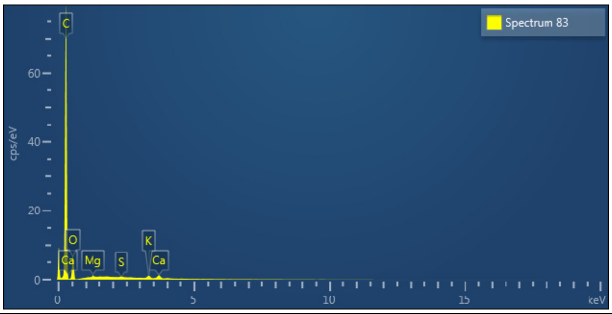

Element	Wt%	Wt% Sigma	Atomic %
C	74.79	0.30	80.36
O	23.66	0.30	19.09
Mg	0.23	0.02	0.12
S	0.11	0.02	0.04
K	0.51	0.03	0.17
Ca	0.69	0.03	0.22
Total:	100.00		100.00

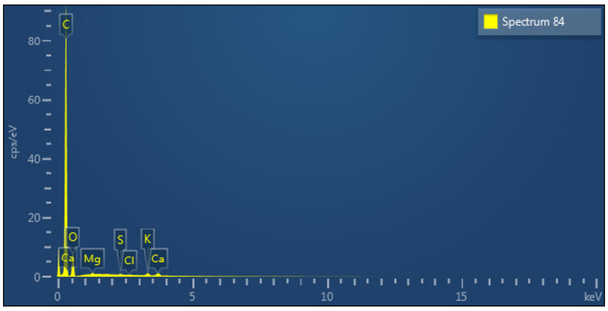

Element	Wt%	Wt% Sigma	Atomic %
C	74.10	0.30	79.70
O	24.54	0.30	19.81
Mg	0.17	0.02	0.09
S	0.11	0.02	0.04
Cl	0.05	0.02	0.02
K	0.46	0.02	0.15
Ca	0.56	0.03	0.18
Total:	100.00		100.00

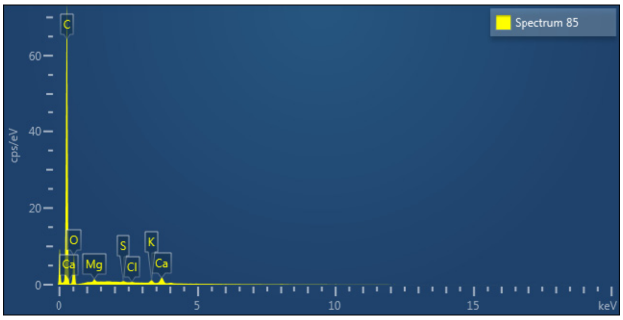

Element	Wt%	Wt% Sigma	Atomic %
C	71.12	0.30	77.41
O	26.65	0.30	21.77
Mg	0.34	0.03	0.18
S	0.12	0.02	0.05
Cl	0.07	0.02	0.02
K	0.54	0.03	0.18
Ca	1.17	0.03	0.38
Total:	100.00		100.00

Table S4: EDX analysis of the spent coffee grounds.

SCG site 1

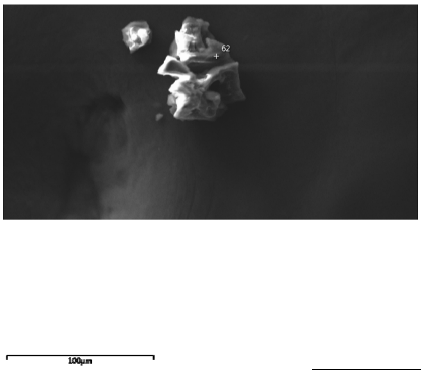

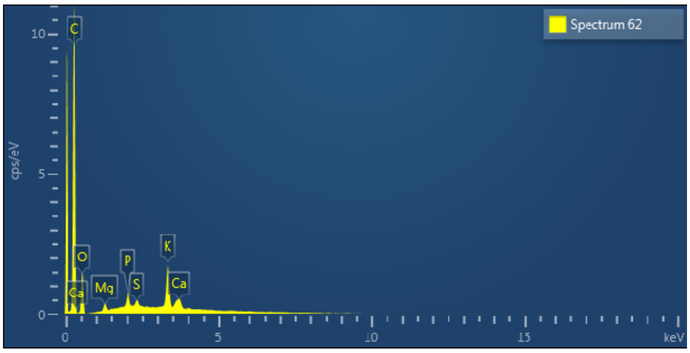

Element	Wt%	Wt% Sigma	Atomic %
**C**	64.48	0.63	73.99
**O**	26.00	0.62	22.40
**Mg**	0.55	0.06	0.31
**P**	1.25	0.07	0.56
**S**	0.52	0.06	0.23
**K**	5.98	0.14	2.11
**Ca**	1.21	0.09	0.42
**Total:**	100.00		100.00

SCG site 2

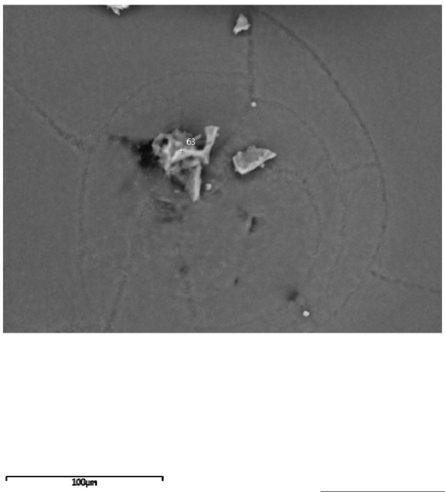

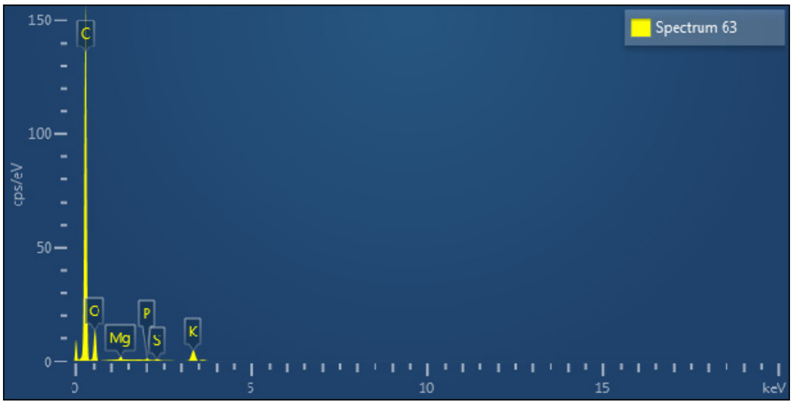

Element	Wt%	Wt% Sigma	Atomic %
**C**	73.18	0.29	79.21
**O**	24.55	0.29	19.95
**Mg**	0.30	0.02	0.16
**P**	0.18	0.02	0.08
**S**	0.13	0.01	0.05

**K**	1.66	0.03	0.55
**Total:**	100.00		100.00

SCG site 3

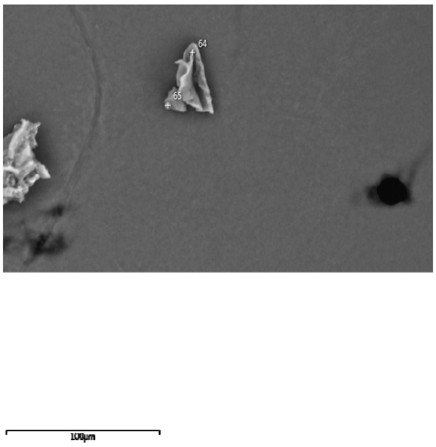

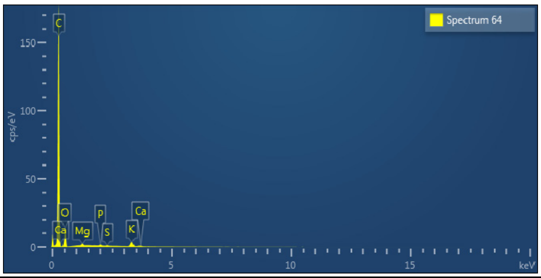

Element	Wt%	Wt% Sigma	Atomic %
**C**	75.44	0.29	81.02
**O**	22.68	0.29	18.29
**Mg**	0.28	0.02	0.15
**P**	0.18	0.02	0.08
**S**	0.11	0.02	0.05
**K**	1.13	0.03	0.37
**Ca**	0.17	0.02	0.05
**Total:**	100.00		100.00

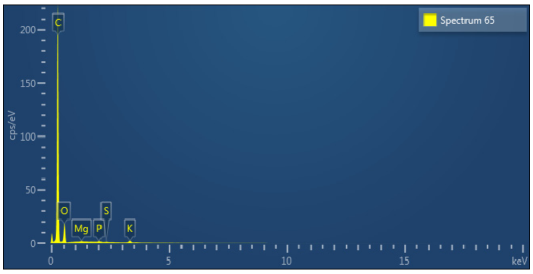

Element	Wt%	Wt% Sigma	Atomic %
**C**	75.20	0.28	80.43
**O**	24.00	0.28	19.27
**Mg**	0.09	0.02	0.05
**P**	0.17	0.02	0.07
**S**	0.05	0.01	0.02
**K**	0.48	0.02	0.16
**Total:**	100.00		100.00

Table S5: Semiquantitative analysis of coffee husk

	%Humidity	%Solubles components	%Hemicellulose	%Cellulose	%Lignin	% ashes
**1**	8.5	38.7	12.7	30.5	17.3	0.08
**2**	9.0	39.8	11.4	32.3	15.6	0.03
**3**	9.0	38.5	12.8	30.1	15.4	0.01
Average	**8.8 ± 0.4**	**39.0 ± 0.7**	**12.3 ± 0.8**	**31 ± 1**	**16 ± 1**	**0.04 ± 0.03**

Table S6: Semiquantitative analysis of coffee parchment

	%Humidity	%Solubles components	%Hemicellulose	%Cellulose	%Lignin	% ashes
**1**	9.0	13.5	13.4	51.6	20.0	0.5

**2**	8.3	11.1	12.1	53.6	20.7	0.4
**3**	7.8	11.9	13.6	46.6	22.7	0.3
Average	**8.8 ±0.6**	**12±1**	**13.0 ± 0.8**	**51 ±4**	**21 ± 1**	**0.4 ± 0.1**

Table S7: Semiquantitative analysis of spent coffee grounds

	%Humidity	%Solubles components	%Hemicellulose	%Cellulose	%Lignin	% ashes
**1**	7.6	23.7	22.1	28.9	22.1	0.08
**2**	6.9	23.5	22.2	29.7	22.2	0.07
**3**	6.3	22.1	23.4	30.4	23.7	0.03
Average	**6.9 ± 0.8**	**23.1 ± 0.9**	**22.6 ± 0.7**	**29.7 ± 0.8**	**22.7 ± 0.9**	**0.06 ± 0.02**

Table S8: Determination of total and insoluble ashes, relative density, apparent density, determination pH and extractives content of coffee residues.

**Total ashes (%)**				
**Samples**	**Replicas**			**Average**
**Coffee husks**	6.37	6.36	6.50	6.41 ± 0.08
**Coffee parchment**	0.80	0.92	1.13	1.0 ± 0.2
**Spent coffee grounds**	2.26	2.22	2.12	2.2 ± 0.1
**Insoluble ashes (%)**				
**Coffee husks**	0.051	0.048	0.052	0.050 ± 0.003
**Coffee parchment**	0.23	0.23	0.26	0.24 ± 0.02
**Spent coffee grounds**	0.081	0.075	0.071	0.076 ± 0.005
**Relative density (kg/m³)**				
**Coffee husks**	563.5	586.7	585.3	579 ± 13
**Coffee parchment**	325.0	320.4	317.5	321 ± 4
**Spent coffee grounds**	523.1	512.0	507.4	514 ± 8
**Apparent density (kg/m³)**				
**Coffee husks**	195.5	204.2	194.2	198 ± 5
**Coffee parchment**	247.6	271.4	288.9	269 ± 21
**Spent coffee grounds**	406.9	423.8	443.0	425 ± 18
**pH**				
**Coffee husks**	4.28	4.26	4.20	4.25 ± 0.04
**Coffee parchment**	4.89	4.90	4.94	4.91 ± 0.03
**Spent coffee grounds**	4.85	4.90	4.86	4.87 ± 0.03
**Extractives content of coffee residues (%)**				
**Coffee husks**	29.4	32.4	30.9	30.9 ± 1.5
**Coffee parchment**	7.0	6.2	7.5	6.9 ± 0.7
**Spent coffee grounds**	28.9	26.2	26.8	27.3 ± 1.4

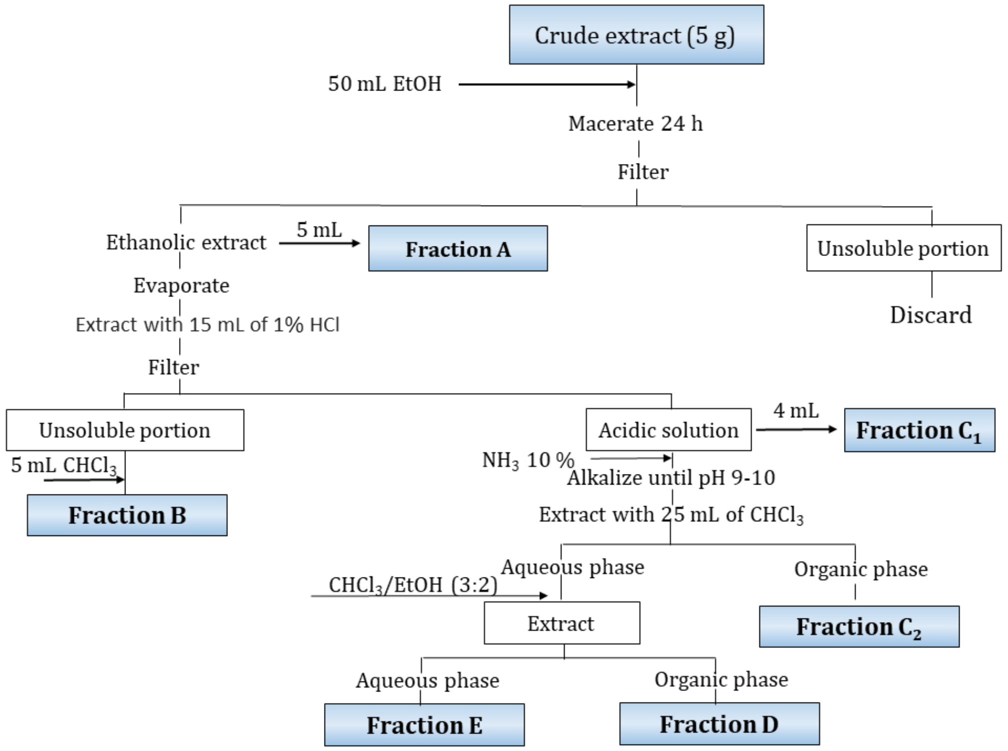

Figure S1: Phytochemical screening
